# Predicting the Need for Renal Replacement Therapy Using a Vascular Occlusion Test and Tissue Oxygen Saturation in Patients in the Early Phase of Multiorgan Dysfunction Syndrome

**DOI:** 10.3390/jcm11051420

**Published:** 2022-03-04

**Authors:** Franz Haertel, Diana Reisberg, Martin Peters, Sebastian Nuding, P. Christian Schulze, Karl Werdan, Henning Ebelt

**Affiliations:** 1 Klinik für Innere Medizin I, Universitaetsklinikum Jena, Am Klinikum 1, 07747 Jena, Germany; christian.schulze@med.uni-jena.de; 2 Klinik für Innere Medizin III, Universitaetsklinikum Halle (Saale), Ernst-Grube-Str. 40, 06120 Halle (Saale), Germany; sunshineblue-88@gmx.de (D.R.); martin.e.peters@gmail.com (M.P.); sebastian.nuding@krankenhaus-halle-saale.de (S.N.); karl.werdan@uk-halle.de (K.W.); henningebelt@googlemail.com (H.E.); 3 Klinik für Pädiatrie, Ameos Klinikum Aschersleben, Eislebener Str. 7A, 06449 Aschersleben, Germany; 4 Klinik für Innere Medizin, Helios Klinikum Jerichower Land, August-Bebel-Str. 55a, 39288 Burg, Germany; 5 Klinik für Innere Medizin II, Krankenhaus “St. Elisabeth”, Mauerstr. 5, 06110 Halle (Saale), Germany; 6 Klinik für Innere Medizin II, Katholisches Krankenhaus “St. Johann Nepomuk”, Haarbergstr. 72, 99097 Erfurt, Germany

**Keywords:** acute kidney injury, tissue oxygen saturation, intensive care, MODS

## Abstract

Background: Acute kidney injury (AKI) is associated with an increased mortality in critically ill patients, especially in patients with multiorgan dysfunction syndrome (MODS). In daily clinical practice, the grading of AKI follows the Kidney Disease: Improving Global Outcomes (KDIGO) criteria. In most cases, a relevant delay occurs frequently between the onset of AKI and detectable changes in creatinine levels as well as clinical symptoms. The aim of the present study was to examine whether a near infrared spectroscopy (NIRS)-based, non-invasive ischemia–reperfusion test (vascular occlusion test (VOT)) together with unprovoked (under resting conditions) tissue oxygen saturation (StO_2_) measurements, contain prognostic information in the early stage of MODS regarding the developing need for renal replacement therapy (RRT). Methods: Within a period of 18 months, patients at the medical intensive care unit of a tertiary university hospital with newly developed MODS (≤24 h after diagnosis, APACHE II score ≥20) were included in our study. The VOT occlusion slope (OS) and recovery slope (RS) were recorded in addition to unprovoked StO_2_. StO_2_ was determined non-invasively in the area of the thenar muscles using a bedside NIRS device. The VOT was carried out by inflating a blood pressure cuff on the upper arm. AKI stages were determined by the changes in creatinine levels, urinary output, and/or the need for RRT according to KDIGO. Results: 56 patients with MODS were included in the study (aged 62.5 ± 14.4 years, 40 men and 16 women, APACHE II score 34.5 ± 6.4). Incidences of the different AKI stages were: no AKI, 16.1% (*n* = 9); AKI stage I, 19.6% (*n* = 11); AKI stage II, 25% (*n* = 14); AKI stage III, 39.3% (*n* = 22). Thus, 39.3% of the patients (*n* = 22) developed the need for renal replacement therapy (AKI stage III). These patients had a significantly higher mortality over 28 days (RRT, 72% (*n* = 16/22) vs. no RRT, 44% (*n* = 15/34); *p* = 0.03). The mean unprovoked StO_2_ of all patients at baseline was 81.7 ± 11.1%, and did not differ between patients with or without the need for RRT. Patients with RRT showed significantly weaker negative values of the OS (−9.1 ± 3.7 vs. −11.7 ± 4.1%/min, *p* = 0.01) and lower values for the RS (1.7 ± 0.9 vs. 2.3 ± 1.6%/s, *p* = 0.02) compared to non-dialysis patients. Consistent with these results, weaker negative values of the OS were found in higher AKI stages (no AKI, −12.7 ± 4.1%/min; AKI stage I, −11.5 ± 3.0%/min; AKI stage II, −11.1 ± 3.3%/min; AKI stage III, −9.1 ± 3.7%/min; *p* = 0.021). Unprovoked StO_2_ did not contain prognostic information regarding the AKI stages. Conclusions: The weaker negative values of the VOT parameter OS are associated with an increased risk of developing AKI and RRT, and increased mortality in the early phase of MODS, while unprovoked StO_2_ does not contain prognostic information in that regard.

## 1. Introduction

Acute kidney injury (AKI) is a life-threatening complication of multiorgan dysfunction syndrome (MODS), and remains a major diagnostic and therapeutic challenge [1,2,3]. It is estimated that approximately 60% of critically ill patients in intensive care units (ICU) are diagnosed with AKI of varying severity [4]. As soon as AKI leads to the need of renal replacement therapy (RRT), hospital mortality increases significantly, to values exceeding 50% [5,6,7], and the subsequent development of chronic kidney disease is possible, even after apparent renal recovery [8].

As a main pathophysiological feature in MODS patients, the impaired tissue metabolism is unable to extract sufficient levels of O_2_, even from an adequate systemic O_2_ supply [9], leading to organ dysfunction or failure, such as in AKI.

In the context of detecting these changes in tissue oxygenation, the concept of tissue oxygen saturation (StO_2_) measured non-invasively via near infrared spectroscopy (NIRS), has gained growing interest. This technology has been used to introduce and characterize new diagnostic parameters. NIRS is an established and reproducible method that allows the continuous monitoring of StO_2_ in real time, and two conditions for measuring StO_2_ have been established: measurements are taken under resting conditions (unprovoked), and using a ischemia–reperfusion test (vascular occlusion test (VOT)) [10,11]. The occlusion and reopening of arterial blood vessels result in blood flow changes that translate into dynamic StO_2_ oscillations. It seems that the analysis of changes in StO_2_ during this circulatory stress test may be more useful for the quantification of MODS-induced microvascular dysfunction [12]; thus further information can, and needs to, be gained.

Studies examining the role of tissue oxygen saturation with respect to renal oxygenation and clinical AKI exist, in particular for infants, but are limited for the adult population. A previously published study on adult cardiac surgery patients by Sakaki et al. demonstrated that tissue oxygen saturation measured in the thumb region has the potential to aid the detection of local renal ischemia [13].

It is important to note that the initial symptoms of AKI are clinically unspecific. Current conventional risk stratification is mainly based on functional blood and urinary markers to determine or estimate kidney injury [14,15]. Having limitations, these methods can complicate the interpretation of results, leading to a delay of the diagnosis and/or misinterpretation of the prognosis. The aim of this study was to investigate the prognostic potential of StO_2_, unprovoked and by means of a VOT, in MODS patients at the time of ICU admission, regarding the need for RRT in AKI.

## 2. Methods

### 2.1. Patients

The actual VOT population was part of the prospective, randomized, controlled MODI_f_Y trial population (EudraCT-Nr.: 2009-015499-88) [16]. The study protocol of the MODI_f_Y trial was approved by the ethics committee of the Martin Luther University of Halle (Saale), Germany. Critically ill patients with newly diagnosed MODS (APACHE II score ≥20) were included in the MODI_f_Y-trial if they had a study-independent indication for invasive hemodynamic monitoring, a sinus rhythm with a heart rate ≥90/min, pre-existing contraindications to beta-blockers, and a signed declaration of informed consent [16]. Exclusion criteria were age <18 years, pregnancy or lactation, patients with chronic renal insufficiency (eGFR < 30 mL/min), malignant hyperthermia, burns, acute rejection after organ transplantation, sick sinus syndrome, third degree sinoatrial or atrioventricular block, cardiac pacemaker, high-grade valvular heart disease, severe hepatic failure, and/or suspected hypoxic brain damage after resuscitation. Patients could be included in the study only within 24 h after diagnosing a MODS, and were prospectively stratified into cardiogenic (cMODS) and septic MODS (sMODS).

### 2.2. Study Endpoint

The clinical endpoint of this post hoc analysis is the need for RRT within 28 days after ICU admission.

### 2.3. Determination and Grading of Acute Kidney Injury

The extent of AKI was determined post hoc by the changes in serum creatinine level, urinary output, and/or the need for RRT in accordance with Kidney Disease: Improving Global Outcomes (KDIGO) criteria [17]. Urinary output (mL/h) was measured every 2 h via an indwelling catheter through the urethra. The body surface area (BSA) was calculated according to the Du Bois and Du Bois formula: BSA = 0.20247 × (Height (m)^0.725^) × (Weight (kg)^0.425^) [18,19].

### 2.4. Renal Replacement Therapy

Continuous venovenous hemodiafiltration (CVVHD) (Multifiltrate^©^, Fresenius Medical Care, St. Wendel, Germany), using citrate anticoagulation was the mode of renal replacement therapy used for all patients. Initiation and management of CVVHD was subject to the discretion of the medical ICU staff. For vascular access, a double or triple lumen catheter, inserted either in the internal jugular or femoral vein, was used. The medical staff was unaware of the results of the VOT parameters, and thus did not make decisions regarding the initiation of RRT upon them.

### 2.5. Unprovoked StO_2_ Measurement and Vascular Occlusion Test

The determination of StO_2_ was performed non-invasively using the NIRS device InSpectra Tissue Spectrometer Model 650 (Hutchinson Technology Inc., Hutchinson, MN, USA) at the time of ICU admission (baseline) and after 96 h (96 h). StO_2_ measurements of any kind, at our intensive care unit, are not part of routine medical care parameters for the treatment of sepsis and/or patients in shock.

The InSpectra StO_2_ system consists of a recording and display monitor, a NIRS glass fiber probe, and a connector. The system is not associated with any relevant burden or hazard to the patient, can be performed at bedside, has a low interobserver variability, and the infrared light used does not cause any physical or chemical changes in the human tissue [11,12]. The probe is positioned in the center axis of the thenar. The system records the StO_2_ of the patient at intervals of 2 s automatically.

The probe emits light with a wavelength of 680–850 nm and a penetration depth of 14–15 mm [11,12,20,21]. Thus, 95% of all signals below the skin surface can be detected [12]. The measurement of StO_2_ is based on spectrophotometric principles that use light absorption to calculate chemical concentrations (Lambert–Beer law). The known specific absorption spectra of oxygenated and deoxygenated hemoglobin allow conclusions to be drawn regarding the oxygen saturation in the tissue. StO_2_ was calculated as follows:

StO_2_ (%) = Oxyhemoglobin/Total hemoglobin × 100

Unprovoked measurement of StO_2_ was obtained immediately after attaching the probe to the patient’s thenar muscle. Values below 75% were considered as hypoperfusion, as recommended by previous studies [22,23,24].

The VOT was performed non-invasively by inflating a blood pressure cuff on the upper arm to 30 mmHg above the systolic blood pressure for 5 min, and deflating immediately after. The parameter describing the phase of desaturation, during this VOT, is referred to as the occlusion slope (OS), and the parameter for the resaturation phase is known as the recovery slope (RS).

The OS reflects the cellular O_2_ consumption under cuff inflation, and steepens with increasing tissue O_2_ metabolism [25]. The OS (%/min) describes the drop in StO_2_ during the VOT from cuff inflation to cuff deflation, and follows a curve with a negative slope. Therefore, the obtained values for OS are always negative. The recovery slope (RS) (%/s) is caused by the immediate return of the blood flow after the relief of the arterial occlusion, and by reactive vasodilatation [25]. The steeper the RS, the faster the tissue is resupplied with O_2_ [25]. The starting point of the recovery slope is ≥5% of the minimum StO_2_, and is measured immediately after the deflation of the pressure cuff until baseline StO_2_ is restored. Reported cut-off values for the OS and RS VOT parameters are −10%/min and 2.7%/s, respectively [26,27,28]. Due to the fact that changes in StO_2_ typically take place slowly during cuff inflation (OS), and rapidly after cuff deflation (RS), different units of OS (%/min) and RS (%/s) are used.

### 2.6. Statistical Analysis

Statistical data analysis was performed using SPSS Statistics (version 26.0, SPSS Inc., IBM, Armonk, New York, NY, USA). Normal distribution was analyzed by Kolmogorov–Smirnov test. Differences in frequency of nominally scaled parameters were compared using Pearson‘s chi-squared test. Metric variables are expressed as mean ± standard deviation, tests for differences between independent and dependent variables were performed using Student’s t-test. Survival was analyzed by Kaplan–Meier statistics, together with a log-rank test. Univariate (unadjusted) as well as multivariate (adjusted) logistic regression models were used to quantify the predictive value of unprovoked StO_2_ and the VOT parameters OS and RS. Receiver operating characteristic (ROC) curves were generated, and their respective area under the curve (AUC) values described. To analyze a possibly significant association between the dependent variables, as well as between the mean values of the StO_2_ parameters within the AKI groups, an analysis of variance (ANOVA) was used. To test for multiple hypotheses, a comparison between the individual AKI groups was based on a post hoc Bonferroni correction. The basis for the test decision was a significance level of *p* < 0.05.

## 3. Results

### 3.1. Patients

StO_2_ parameters were analyzed in 56 critically ill patients. Table 1 lists the baseline characteristics according to the patients’ RRT status. Mean unprovoked StO_2_ at baseline was 81.7 ± 11.1% for all patients. Only nine patients (16.1%) had a pathological, unprovoked StO_2_ below 75%. A total of 52 patients (92.9%) were intubated and received an invasive mechanical ventilation, with an average FiO_2_ of 70.6 ± 20.3% and mean arterial pO_2_ of 15.1 ± 6.9 kPa, with no difference between the two groups (Table 1).

### 3.2. Changes in Kidney Function

Regarding changes in kidney function, we observed the frequencies displayed in Table 1. RRT was started in 39.3% (22/56) of all patients, and in 39% (16/41) and 40% (6/15) of patients in the sMODS and cMODS subgroups, respectively, within 28 days after ICU admission. The mean interval between ICU admission and the initiation of RRT was 43.3 ± 41.3 h, with 77.7% of the patients (*n* = 17) starting dialysis within 48 h. All patients receiving RRT were included in AKI stage III. These patients had a higher mortality within the following 28 days (RRT, 72% (*n* = 16/22) vs. no RRT, 44% (*n* = 15/34); *p* = 0.03). The decision for the initiation of RRT was based on the discretion of the medical ICU staff, involving multifactorial clinical considerations. Altered and/or decreased urine output was the first and major signal of renal function loss. Urine output for patients receiving RRT in this collective was variable, ranging from anuria to normal, or even above normal levels. The most common reasons for urinary output being compromised were anuria (45.5%, *n* = 10/22), oliguria (27.3%, *n* = 6/22), and polyuria (4.5%, *n* = 1/22). Five patients (22.7%) with normal urinary output receiving RRT were admitted to the ICU with relevantly elevated renal retention markers (e.g., serum creatinine, blood urea nitrogen).

### 3.3. Association between Tissue Oxygen Saturation and Renal Replacement Therapy

**Figure 1:**
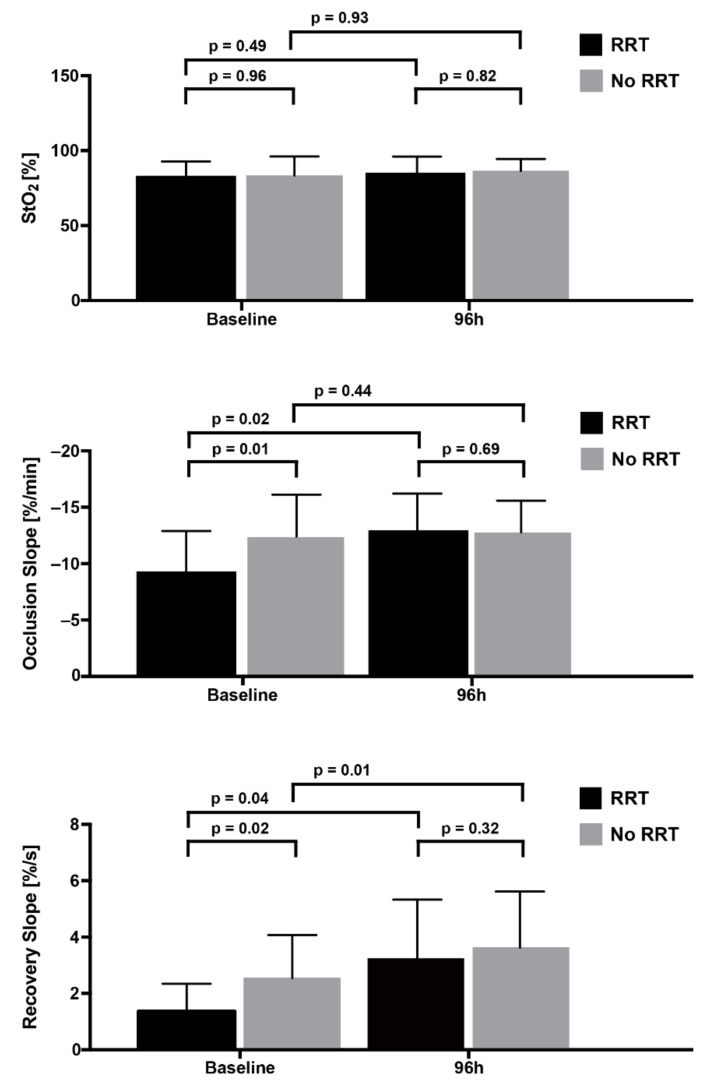
Unprovoked StO_2_ and the VOT occlusion and recovery slopes at baseline and after 96 h, in the context of renal replacement therapy (RRT) status until day 28.

Figure 1 shows the unprovoked StO_2_ and VOT parameters according to RRT status. Patients not receiving RRT had significantly better (more negative) OS values and better (more positive) RS values at baseline compared to dialysis patients. After 96 h, these differences can still be observed, albeit not reaching statistical significance. In general, these values improved under standard ICU therapy within 96 h, regardless of RRT status. For unprovoked StO_2_, no differences could be observed in this regard; unprovoked StO_2_ tended to remain almost unchanged throughout the ICU stay. RRT rates did not differ between patients with a pathologically low unprovoked StO_2_, i.e., below 75% (RRT, 44.4% (*n* = 4/9)) and patients with an unprovoked StO_2_, i.e., within normal range of above 75% (RRT, 38.3%; (*n* = 18/47); *p* = 0.51).

**Figure 2:**
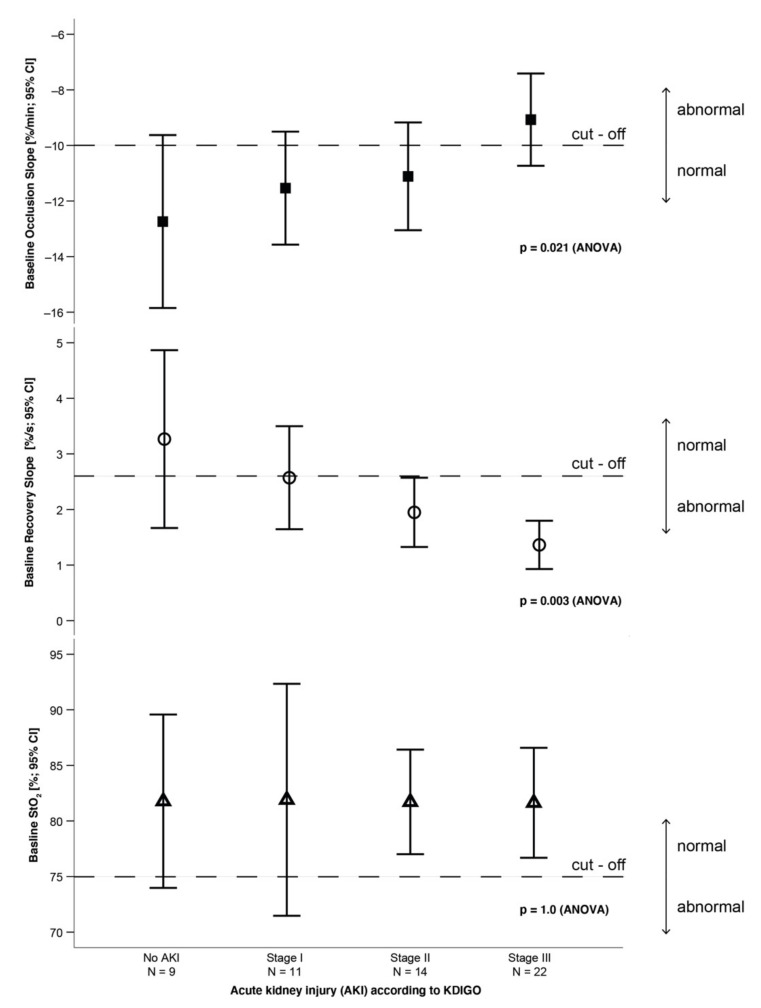
Mean ± standard deviation for unprovoked StO_2_ and the VOT parameters OS and RS at baseline, regarding the stages of acute kidney injury (AKI) according to Kidney Disease: Improving Global Outcomes (KDIGO) criteria. Bonferroni correction for OS: no AKI vs. AKI stage III, *p* = 0.036; no AKI vs. AKI stage I/AKI stage II, *p* > 0.05; AKI stage I vs. AKI stage II/AKI stage III, *p* > 0.05. Bonferroni correction for RS: no AKI vs. AKI stage III, *p* = 0.007; no AKI vs. AKI stage I/AKI stage II, *p* > 0.05; AKI stage I vs. AKI stage II/AKI stage III, *p* > 0.05. Bonferroni correction for unprovoked StO_2_: no AKI vs. AKI stage III, *p* > 0.05; no AKI vs. AKI stage I/AKI stage II, *p* > 0.05; AKI stage I vs. AKI stage II/AKI stage III, *p* > 0.05.

**Figure 3:**
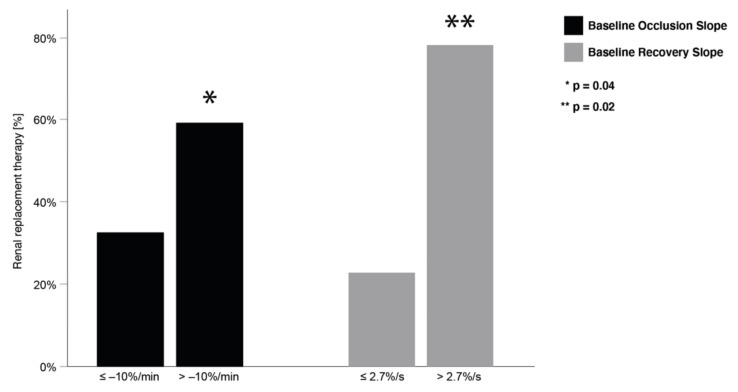
Percentage of patients developing the need for renal replacement therapy in relation to their VOT occlusion slope at baseline; *p* = 0.045.

As shown in Figure 2, the occlusion and recovery slopes were more impaired in the advanced stages of AKI. For all stages of AKI, unprovoked StO_2_ remained stable. The proportion of patients with dialysis requiring AKI was significantly greater in the groups with weaker occlusion and recovery slopes (Figure 3).

**Figure 4:**
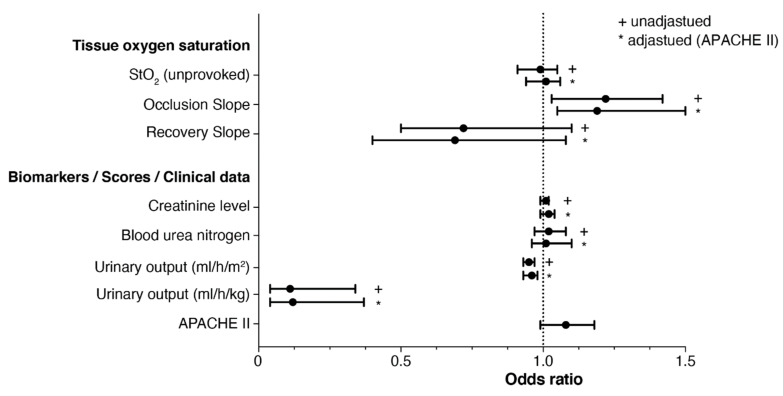
Forrest plot showing the unadjusted and adjusted odds ratios regarding the prediction of renal replacement therapy. APACHE II, acute physiology and chronic health evaluation.

A binary logistic regression model was used to calculate the odds ratios regarding the need for RRT (Figure 4). After adjusting the VOT parameters for the APACHE II score, results only remained significant for the occlusion slope. Weaker negative OS values were therefore associated with higher risk of need for RRT. The recovery slope did not provide a prediction for the development of the need for RRT; however, a tendency can be inferred.

Using ROC curves (Table 2), relevant prognostic information regarding RRT were found for OS (AUC, 0.70; *p* = 0.04). Serum creatinine and blood urea nitrogen levels at baseline showed no significant AUCs, and only urinary output was the strongest clinical predictor for RRT (Table 2).

**Figure 5:**
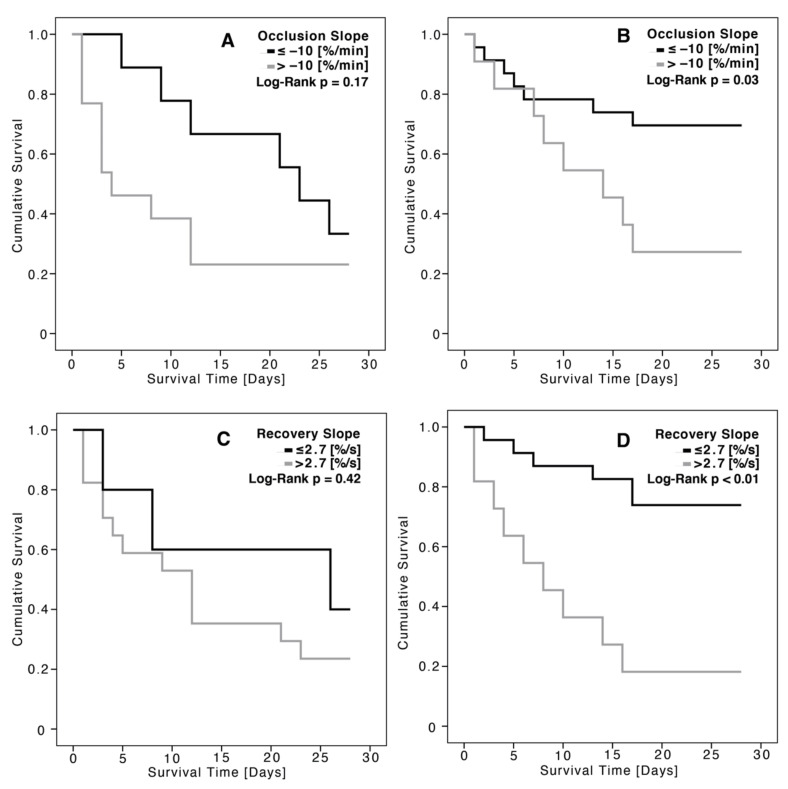
The VOT occlusion slopes at baseline regarding 28-day mortality for patients receiving RRT (**A**) and patients not needing RRT (**B**), as well as the VOT recovery slopes at baseline regarding 28-day mortality for patients receiving RRT (**C**) and patients not needing RRT (**D**), using Kaplan–Meier survival curves; level of significance measured by *p*.

Figure 5 shows the Kaplan–Meier survival curves based on RRT status and the previously mentioned cut-off values for the VOT parameters. Patients with unimpaired VOT parameters that did not need RRT had the highest survival rate.

## 4. Discussion

To date, changes in StO_2_ have not been investigated in patients at the early stage of MODS regarding to predict the development of acute kidney injury and the need for renal replacement therapy during ICU treatment.

### 4.1. Prognostic Relevance of Unprovoked StO_2_ Regarding Renal Replacement Therapy

Critically ill patients with a pathological baseline StO_2_ (<75%) did not require RRT more often than patients with a baseline StO_2_ (>75%). Observing the ROC curve and binary logistic regression, unprovoked StO_2_ remains unsuitable as a prognostic marker for predicting the necessity for RRT, according to our data. We conclude that no prognostic relevance for unprovoked StO_2_ could be determined in the present work for patients in the early stages of MODS.

Regardless of our own findings, there are data available in the literature, that suggest that unprovoked StO_2_ can be used to supplement AKI prediction, although these studies were not conducted in MODS patients.

Choi et al. [29] assessed, in a prospective clinical study, the significance of unprovoked StO_2_ for the prediction of postoperative AKI using transcutaneous NIRS (INVOS 5100C; Somanetics Co., Troy, MI, USA). They used the technique intraoperatively, rather than postoperatively, in 95 cardiac surgery patients directly with transcutaneous measurements taken in the area of the kidneys. Of these patients, 35.8% postoperatively developed AKI. The onset of postoperative AKI was significantly predicted by pathologic StO_2_-values measured intraoperatively prior to a relevant increase in serum creatinine. In this regard, a study by Owens et al. [30] on pediatric patients undergoing cardiac surgery demonstrates that the StO_2_ measured at the forehead using NIRS (INVOS 5100B; Somanetics Co., Troy, MI, USA) appears to correlate with renal dysfunction (AKI), decreased systemic oxygen delivery, and the overall postoperative course. A previously mentioned study by Sakaki et al. [13] involved patients undergoing cardiopulmonary bypass surgery, alongside a StO_2_ measuring set up using NIRS (INVOS 5100 C; Medtronic, Minneapolis, MN, USA) that included sensors placed on three different regions, such as the patients’ forehead, abdomen, and the lateral side of the thigh, during the induction of anesthesia. StO_2_ measurements derived from these regions revealed that an impaired regional oxygen saturation (unprovoked) at the thigh region of ≤67% was predictive of acute kidney injury within 24 h after surgery.

In a recent publication by Harer et al. [31] on conducting StO_2_ measurements in neonates, these authors established the notion that the use of direct renal tissue oxygenation monitoring could be used to improve renal outcomes. It holds significant promise to function as a real time, early indicator of poor renal perfusion, which may help with the development of specific treatment protocols to prevent or decrease the severity of AKI [31]. The current paradigm includes an event of renal tissue injury, followed by a relevantly delayed decrease in urine output/increase in serum creatinine. Thus, markers of AKI start to change 12–48 h after irreversible damage has already occurred, and may signal permanent tissue injury [31]. Changes in StO_2_, on the other hand, may reflect an earlier time period when renal ischemia is still reversible and responsive to fluid management, transfusions, inotropic support, or medication administration that may resolve ongoing injury [31].

These findings suggest not only the possibility of integrating StO_2_ into patient monitoring, but also the importance of research concerning the location of measured tissue oxygen saturations for estimating function vs. dysfunction. However, we support an additive benefit of NIRS-derived StO_2_ to traditional renal function parameters, and it must be highlighted in this regard that local measurements of StO_2_ in the regions of the kidneys have to appreciate the heterogeneity in the metabolic requirements within the kidneys, with the medulla being relatively hypoxic compared to the cortex [32].

### 4.2. Prognostic Relevance of StO_2_ under VOT Regarding Renal Replacement Therapy

In the present study, 39.3% of patients with MODS developed an AKI requiring RRT. These patients had a significantly higher mortality rate over 28 days (non-survivors vs. survivors, 72% vs. 44%, *p* < 0.05). The presented data show that especially the VOT occlusion slope could help in the prediction of the need for RRT. Generally, data for occlusion slope values predicting RRT are limited in the literature; therefore, at present, only analogies can be drawn.

AKI possesses components of ischemia–reperfusion injury, direct inflammatory injury, coagulation, and endothelial cell dysfunction and apoptosis [33]. Recent evidence revealed differences in the pathophysiologic mechanisms between sepsis-induced AKI and non-septic AKI [33].

According to the work of Fink et al. [34,35], one of the major causes of organ dysfunction (e.g., AKI) in MODS patients is the impairment of oxygen utilization by pathologies of mitochondrial function (cytopathic hypoxia). This means that the VOT parameters of StO_2_ are primarily to be understood as an expression of oxygen utilization at the cellular level. Since the impairment of oxygen utilization also occurs in organ systems of the splanchnic area in MODS with a shunting of the blood circulation in the periphery and in the splanchnic area, where the blood flow is diverted from less vital to absolutely vital organ systems [36]. This could explain the fact that the above-mentioned VOT parameters are able to indirectly predict an AKI with need for RRT. Measuring StO_2_ using a VOT has therefore more value for predicting the need for RRT, as it allows a functional characterization of tissue as opposed to unprovoked StO_2_ which only provides static parameters.The work of Singh et al. [37] shows a correlation between GFR and renal oxygen consumption in patients with early AKI after surgery; patients developing AKI tend to have a steeper correlation curve than healthy controls. Renal oxygen extraction was then found to be higher in AKI patients compared to healthy controls, without any significant differences regarding cardiac index and mean arterial pressure. They also demonstrated that the relationship between glomerular filtration rate (GFR) and renal oxygen consumption in clinical ischemic AKI is accompanied by a severe impairment of the renal oxygen demand/supply relationship. They found a 70% higher renal oxygen extraction in the presence of pronounced vasoconstriction and hypoperfusion compared to controls.

The results of our study, that VOT-derived parameters of StO_2_ are superior to unprovoked StO_2_ alone in predicting RRT, are reinforced by the recent work of Chaves et al. [38], also using NIRS (InSpectra Tissue Spectrometer model 650; Hutchinson Technology Inc., Hutchinson, MN, USA). The study by Chaves et al. included a population of nine patients (77.8% men, 66.7% sepsis-induced AKI) admitted to ICU due to non-surgical reasons, and urinary output immediately before the initiation of RRT was zero in all cases. A drop in minimum StO_2_ values during the VOT compared to baseline in the first 24 h after RRT initiation was the only significant change in this study. This association remained significant after adjustment for baseline disease severity. The baseline occlusion slope before the initiation of dialyses showed a pathologically reduced median value of −8.3 (4.4–10.4)%/min, similar to our baseline occlusion slope of −9.1 (±3.7)%/min of patients that receive RRT. The baseline recovery slope of their study showed a median of 1.6 (1.2–3.1)%/s, which is also similar to the baseline recovery slope of our RRT collective of 1.7 (±0.9)%/s. The assessment of near infrared spectroscopy-derived parameters immediately before, and 1, 4, and 24 h after the initiation of CVVHD showed an improvement in the VOT parameters, although only a slight tendency could be inferred. These findings suggest higher oxygen consumption during the first 24 h of RRT, as minimum StO_2_ during the VOT is thought to be an indicator of the extent of ischemia [38,39].

Since there have been no further studies for patients that focus specifically on AKI and additionally using a VOT, our results cannot be compared or evaluated with broader pre-existing research.

### 4.3. Limitations of This Study, StO_2_ Measurement, and VOT

Due to the design of the MODIfY study, there are no control patients that would allow a comparison between MODS patients and ICU patients without MODS or even healthy controls. All data were derived from a single-center study, and the decision to initiate RRT was not based on a specific protocol, but left to the clinical judgement of the treating physicians. According to our data, impaired urine output was the main reason for RRT in the analysis, which is part of the KDIGO criteria. The need for RRT initiation can develop from multiple medical problems that evolve during the ICU stay and impact kidney function, and must not be related to the onset of MODS, but can also be the result of, for example, co-medication with nephrotoxic potential [40].

Creatinine level (μmol/L) should have a strong predictability to develop AKI, although the mere requirement of RRT is the primary outcome of our study. Comparing the predictability of RRT, we observed that relevant changes in the occlusion slope (>−10%/min) at the time of ICU admission are superior to creatinine level. However, this is not surprising, as there is a diagnostic window between onset of kidney injury and subsequent changes in serum creatinine. The VOT as a functional test, however, reveals information, in real time, on oxygen consumption and microvasculature reactivity as a means to assess microvascular reserve and tissue integrity in just a few minutes; correlations of abnormal VOT parameters with creatinine are not available in literature yet [11,41]. However, urinary output alone showed the strongest prognostic value for the need of RRT, both in the ROC curve analysis and after adjusted logistic regression. The addition of urine output to serum creatinine provides the strongest clinical method for identification, and therefore earlier recognition of AKI in need of RRT [17]. As known from previous studies, a substantial rise in creatinine will only occur when AKI has already developed to a clinically apparent degree [17,42]. A decreased urine output may be the first signal of significant renal function before serum creatinine will reach AKI criteria for advanced stages. However, published study results that focus on clinical markers, such as creatinine and urine output, show large heterogeneity, making them hard to compare to one another due to the characteristics of patients included, the AKI definition or criteria used, reference or baseline serum creatinine used, the time frame over which AKI was assessed, and whether/how urine output was used/determined [42]. For example, the current definition formulated by KDIGO is very similar to the AKI definition, but the time frame was extended from 48 h to 7 days, leading to increased AKI incidences [17,42].

Overall, we included various MODS patients; in this regard, the mechanism of development of AKI must be considered multifactorial. Our methods might be associated with tissue perfusion and oxygen delivery; however, some may assume that this may be the surrogate of severity of illness, thus will not be associated with the risk of AKI independently. However, as this is a small study, there is not enough power to assess the independent associations with adjusting multiple factors, not just APACHE II score.

NIRS may be affected by the amount of adipose tissue, and by the presence of edema at the assessment site, which is a common finding in ICU patients [43]. Furthermore, changes detected in this study may have been artificially caused by RRT itself, as dialysis may induce pre-capillary sphincter constriction due to changes in electrolyte concentrations, and a drop in core temperature in response to dialysis against cool dialysate [38,44]. When comparing our results to previous studies, it is worth mentioning that a variety of devices used to measure StO_2_ via NIRS have become commercially available over the last decade. So far, there is no general agreement regarding a standard for vascular occlusion testing, which might limit the comparability of our results with other studies. For this work, VOT was performed with a five minute cuff compression, as such an approach with a fixed compression time has been preferred in the past [45,46].

## 5. Conclusions

The present study shows that vascular occlusion testing using tissue oxygen saturation (StO_2_) allows risk stratification of patients in the early phase of MODS, and is associated with acute kidney injury and increased mortality within 28 days. Therefore, StO_2_ together with a VOT could be considered as a non-invasive method to supplement the monitoring of MODS in critically ill patients in order to close the therapeutic window, thus reversing or preventing further kidney injury. Unprovoked StO_2_ does not provide any relevant implications regarding a foreseeable RRT.

## Figures and Tables

**Table 1:** Baseline data of the total study population regarding RRT status.

	Study Population (*N* = 56)		
	No RRT	RRT	*p*-Value
	(*N* = 34)	(*N* = 22)	
**Demographics**			
Age (years, mean ± SD)	64.5 ± 15.0	59.4 ± 13.2	n.s.
Male (*N*, %)	25 (73.5)	15 (68.2)	n.s.
Female (*N*, %)	9 (26.5)	7 (31.8)	n.s.
BMI (kg/m^2^, mean ± SD)	27.6 ± 8.9	24.9 ± 4.6	n.s.
BSA (m^2^, mean ± SD)	1.96 ± 0.29	1.91 ± 0.21	n.s.
<70 years (*N*, %)	18 (52.9)	16 (47.1)	n.s.
≥70 years (*N*, %)	16 (47.1)	6 (27.3)	n.s.
**Tissue oxygen saturation parameters**			
StO_2_ unprovoked (%, mean ± SD)	82 ± 10.8	82.1 ± 10.9	n.s.
Occlusion slope (%/min, mean ± SD)	−11.7 ± 4.1	−9.1 ± 3.7	0.02
Recovery slope (%/s, mean ± SD)	2.3 ± 1.6	1.7 ± 0.9	0.01
**Clinical features**			
APACHE II score (mean ± SD)	33.4 ± 6.2	36.3 ± 6.4	n.s.
Creatinine level (µmol/L)	157.9 ± 77.0	209. 9 ± 121.3	n.s.
BUN (mmol/L, mean ± SD)	13.8 ± 10.2	16.4 ± 8.6	n.s.
Urinary output (mL/h/m^2^ BSA, mean ± SD)	53.4 ± 14.9	21.6 ± 47.1	0.001
Urinary output (ml/h/kg, mean ± SD)	1.3 ± 0.2	0.5 ± 1.0	<0.001
Body temperature (°C, mean ± SD)	37.0 ± 1.4	36.9 ± 1.3	n.s.
CRP (mg/L, mean ± SD)	186.0 ± 144.1	258.2 ± 173.4	n.s.
Invasive mechanical ventilation (*N*, %)	32 (94.1)	20 (90.1)	n.s.
SpO_2_ (%, mean ± SD)	96.2 ± 5.8	95.1 ± 6.5	n.s.
FiO_2_ (%, mean ± SD)	73.2 ± 21.2	67.9 ± 19.3	n.s.
pO_2_ (kPa, mean ± SD)	15.7 ± 7.4	14.6 ± 4.9	n.s.
Time of MODS diagnosis relative to ICU admission (h, mean ± SD)	35.4 ± 31.6	18.2 ± 13.1	0.03
Haemoglobin (mmol/L, mean ± SD)	6.9 ± 1.2	6.4 ± 1.3	n.s.
Relative norepinephrine dose (μg/kg/min, mean ± SD)	0.46 ± 0.06	0.59 ± 0.12	n.s.
Relative doputamine dose (μg/kg/min, mean ± SD)	3.1 ± 0.5	4.3 ± 0.9	n.s.
**AKI according to KDIGO**			
No AKI (*N*, %)	9 (26.5)	0 (0)	<0.001
Stage I (*N*, %)	11 (32.4)	0 (0)	<0.001
Stage II (*N*, %)	14 (41.1)	0 (0)	<0.001
Stage III (*N*, %)	0 (0)	22 (100)	<0.001
**Type of MODS**			
Cardiogenic MODS (*N*, %)	9 (26.5)	6 (27.3)	n.s.
Septic MODS (*N*, %)	25 (73.5)	16 (72.7)	n.s.
**Comorbidities**			
Hypertension (*N*, %)	17 (50)	7 (31.8)	n.s.
Diabetes (*N*, %)	12 (35.3)	5 (22.7)	n.s.
CKD (*N*, %)	3 (8.8)	3 (13.6)	n.s.
Past myocardial infarction (*N*, %)	7 (20.6)	5 (22.7)	n.s.
Past stroke (*N*, %)	1 (2.9)	1 (4.5)	n.s.
Active malignancy (*N*, %)	7 (20.6)	4 (18.2)	n.s.

AKI, acute kidney injury; KDIGO, Kidney Disease: Improving Global Outcomes; APACHE II score, acute physiology and chronic health evaluation II score; BMI, body mass index; CRP, C-reactive protein; MODS, multiorgan dysfunction syndrome; CKD, chronic kidney disease; ICU, intensive care unit; RRT, renal replacement therapy; SpO_2_, peripheral oxygen saturation (measured by pulse oximetry); N, number of patients; n.s., not significant; BSA, body surface area; h, hour; BUN, blood urea nitrogen; *p* < 0.05, statistically significant.

**Table 2:** Unprovoked StO_2_, VOT parameters, APACHE II score, and creatinine level at baseline and their respective AUC values, regarding the prediction of renal replacement therapy.

	AUC	CI (95%)	*p*-Value
**Tissue oxygen saturation parameters**			
StO_2_, unprovoked	0.52	0.36–0.67	n.s.
Occlusion slope	0.70	0.54–0.84	0.04
Recovery slope	0.59	0.44–0.74	n.s.
**Clinical parameters**			
Creatinine level	0.64	0.47–0.83	n.s.
Blood urea nitrogen	0.61	0.48–0.74	n.s.
Urinary output	0.94	0.85–1.0	<0.001
APACHE II score	0.63	0.47–0.78	n.s.

APACHE II score, acute physiology and chronic health evaluation II score; AUC, area under the curve; CI, confidence interval; VOT, vascular occlusion test; n.s., not significant; *p* < 0.05, statistically significant.

## References

[1] Brivet F.G., Kleinknecht D.J., Loirat P., Landais P.J. (1996). Acute renal failure in intensive care units—Causes, outcome, and prognostic factors of hospital mortality; a prospective, multicenter study. French Study Group on Acute Renal Failure. Crit. Care Med..

[2] D’Avila D.O., Cendoroglo Neto M., dos Santos O.F., Schor N., Poli de Figueiredo C.E. (2004). Acute renal failure needing dialysis in the intensive care unit and prognostic scores. Ren. Fail..

[3] Guerin C., Girard R., Selli J.M., Ayzac L. (2002). Intermittent versus continuous renal replacement therapy for acute renal failure in intensive care units: Results from a multicenter prospective epidemiological survey. Intensive Care Med..

[4] Hoste E.A., Bagshaw S.M., Bellomo R., Cely C.M., Colman R., Cruz D.N., Edipidis K., Forni L.G., Gomersall C.D., Govil D. (2015). Epidemiology of acute kidney injury in critically ill patients: The multinational AKI-EPI study. Intensive Care Med..

[5] Karsou S.A., Jaber B.L., Pereira B.J. (2000). Impact of intermittent hemodialysis variables on clinical outcomes in acute renal failure. Am. J. Kidney Dis..

[6] Liano F., Junco E., Pascual J., Madero R., Verde E. (1998). The spectrum of acute renal failure in the intensive care unit compared with that seen in other settings. The Madrid Acute Renal Failure Study Group. Kidney Int. Suppl..

[7] McCarthy J.T. (1996). Prognosis of patients with acute renal failure in the intensive-care unit: A tale of two eras. Mayo Clin. Proc..

[8] Jones J., Holmen J., De Graauw J., Jovanovich A., Thornton S., Chonchol M. (2012). Association of complete recovery from acute kidney injury with incident CKD stage 3 and all-cause mortality. Am. J. Kidney Dis..

[9] Gustot T. (2011). Multiple organ failure in sepsis: Prognosis and role of systemic inflammatory response. Curr. Opin. Crit. Care.

[10] Gomez H., Mesquida J., Simon P., Kim H.K., Puyana J.C., Ince C., Pinsky M.R. (2009). Characterization of tissue oxygen saturation and the vascular occlusion test: Influence of measurement sites, probe sizes and deflation thresholds. Crit. Care.

[11] Lipcsey M., Woinarski N.C., Bellomo R. (2012). Near infrared spectroscopy (NIRS) of the thenar eminence in anesthesia and intensive care. Ann. Intensive Care.

[12] Creteur J. (2008). Muscle StO_2_ in critically ill patients. Curr. Opin. Crit. Care.

[13] Sakaki K., Kitamura T., Kohira S., Torii S., Mishima T., Hanayama N., Kobayashi K., Ohkubo H., Miyaji K. (2020). Regional thigh tissue oxygen saturation during cardiopulmonary bypass predicts acute kidney injury after cardiac surgery. J. Artif. Organs.

[14] Makris K., Spanou L. (2016). Acute Kidney Injury: Definition, Pathophysiology and Clinical Phenotypes. Clin. Biochem. Rev..

[15] Makris K., Spanou L. (2016). Acute Kidney Injury: Diagnostic Approaches and Controversies. Clin. Biochem. Rev..

[16] Nuding S., Ebelt H., Hoke R.S., Krummenerl A., Wienke A., Muller-Werdan U., Werdan K. (2011). Reducing elevated heart rate in patients with multiple organ dysfunction syndrome by the I (f) (funny channel current) inhibitor ivabradine: MODI (f)Y trial. Clin. Res. Cardiol..

[17] Mizuno T., Sato W., Ishikawa K., Shinjo H., Miyagawa Y., Noda Y., Imai E., Yamada K. (2012). KDIGO (Kidney Disease: Improving Global Outcomes) criteria could be a useful outcome predictor of cisplatin-induced acute kidney injury. Oncology.

[18] Burton R.F. (2008). Estimating body surface area from mass and height: Theory and the formula of Du Bois and Du Bois. Ann. Hum. Biol..

[19] Du Bois D., Du Bois E.F. (1989). A formula to estimate the approximate surface area if height and weight be known. 1916. Nutrition.

[20] Murkin J.M., Arango M. (2009). Near-infrared spectroscopy as an index of brain and tissue oxygenation. Br. J. Anaesth..

[21] Smith A.M., Mancini M.C., Nie S. (2009). Bioimaging: Second window for in vivo imaging. Nat. Nanotechnol..

[22] Carlile C., Wade C.E., Baraniuk M.S., Holcomb J.B., Moore L.J. (2015). Evaluation of StO_2_ tissue perfusion monitoring as a tool to predict the need for lifesaving interventions in trauma patients. Am. J. Surg..

[23] Cohn S.M., Nathens A.B., Moore F.A., Rhee P., Puyana J.C., Moore E.E., Beilman G.J. (2007). Tissue oxygen saturation predicts the development of organ dysfunction during traumatic shock resuscitation. J. Trauma.

[24] Crookes B.A., Cohn S.M., Bloch S., Amortegui J., Manning R., Li P., Proctor M.S., Hallal A., Blackbourne L.H., Benjamin R. (2005). Can near-infrared spectroscopy identify the severity of shock in trauma patients?. J. Trauma.

[25] Lipcsey M., Eastwood G.M., Woinarski N.C., Bellomo R. (2012). Near-infrared spectroscopy of the thenar eminence: Comparison of dynamic testing protocols. Crit. Care Resusc. J. Australas. Acad. Crit. Care Med..

[26] Gomez H., Torres A., Polanco P., Kim H.K., Zenker S., Puyana J.C., Pinsky M.R. (2008). Use of non-invasive NIRS during a vascular occlusion test to assess dynamic tissue O_2_ saturation response. Intensive Care Med..

[27] Luengo C., Resche-Rigon M., Damoisel C., Kerever S., Creteur J., Payen D. (2013). Comparison of two different generations of “NIRS” devices and transducers in healthy volunteers and ICU patients. J. Clin. Monit. Comput..

[28] Mayeur C., Campard S., Richard C., Teboul J.L. (2011). Comparison of four different vascular occlusion tests for assessing reactive hyperemia using near-infrared spectroscopy. Crit. Care Med..

[29] Choi D.K., Kim W.J., Chin J.H., Lee E.H., Don Hahm K., Yeon Sim J., Cheol Choi I. (2014). Intraoperative renal regional oxygen desaturation can be a predictor for acute kidney injury after cardiac surgery. J. Cardiothorac. Vasc. Anesth..

[30] Owens G.E., King K., Gurney J.G., Charpie J.R. (2011). Low renal oximetry correlates with acute kidney injury after infant cardiac surgery. Pediatric Cardiol..

[31] Harer M.W., Chock V.Y. (2020). Renal Tissue Oxygenation Monitoring-An Opportunity to Improve Kidney Outcomes in the Vulnerable Neonatal Population. Front. Pediatr..

[32] Bullen A., Liu Z.Z., Hepokoski M., Li Y., Singh P. (2017). Renal Oxygenation and Hemodynamics in Kidney Injury. Nephron.

[33] Majumdar A. (2010). Sepsis-induced acute kidney injury. Indian J. Crit. Care Med..

[34] Fink M. (1997). Cytopathic hypoxia in sepsis. Acta Anaesthesiol. Scandinavica. Suppl..

[35] Fink M.P. (2001). Cytopathic hypoxia. Mitochondrial dysfunction as mechanism contributing to organ dysfunction in sepsis. Crit. Care Clin..

[36] Gruartmoner G., Mesquida J., Masip J., Martinez M.L., Villagra A., Baigorri F., Pinsky M.R., Artigas A. (2014). Thenar oxygen saturation during weaning from mechanical ventilation: An observational study. Eur. Respir. J..

[37] Singh P., Ricksten S.E., Bragadottir G., Redfors B., Nordquist L. (2013). Renal oxygenation and haemodynamics in acute kidney injury and chronic kidney disease. Clin. Exp. Pharmacol. Physiol..

[38] Chaves R.C.F., Tafner P., Chen F.K., Meneghini L.B., Correa T.D., Rabello Filho R., Cendoroglo Neto M., Santos O., Serpa Neto A. (2019). Near-infrared spectroscopy parameters in patients undergoing continuous venovenous hemodiafiltration. Einstein (Sao Paulo).

[39] Bezemer R., Lima A., Myers D., Klijn E., Heger M., Goedhart P.T., Bakker J., Ince C. (2009). Assessment of tissue oxygen saturation during a vascular occlusion test using near-infrared spectroscopy: The role of probe spacing and measurement site studied in healthy volunteers. Crit. Care.

[40] Patel J.B., Sapra A. (2022). Nephrotoxic Medications.

[41] Tafner P., Chen F.K., Rabello R.F., Correa T.D., Chaves R.C.F., Serpa A.N. (2017). Recent advances in bedside microcirculation assessment in critically ill patients. Rev. Bras. Ter. Intensiva.

[42] Koeze J., Keus F., Dieperink W., van der Horst I.C., Zijlstra J.G., van Meurs M. (2017). Incidence, timing and outcome of AKI in critically ill patients varies with the definition used and the addition of urine output criteria. BMC Nephrol..

[43] Uhle F., Lichtenstern C., Weigand M.A., Schuster H.P., Müller Werdan U., Werdan K. (2016). Pathophysiologie. Sepsis und MODS.

[44] Pipili C., Vasileiadis I., Grapsa E., Tripodaki E.S., Ioannidou S., Papastylianou A., Kokkoris S., Routsi C., Politou M., Nanas S. (2016). Microcirculatory alterations during continuous renal replacement therapy in ICU: A novel view on the ‘dialysis trauma’ concept. Microvasc. Res..

[45] Creteur J., Carollo T., Soldati G., Buchele G., De Backer D., Vincent J.L. (2007). The prognostic value of muscle StO_2_ in septic patients. Intensive Care Med..

[46] Payen D., Luengo C., Heyer L., Resche-Rigon M., Kerever S., Damoisel C., Losser M.R. (2009). Is thenar tissue hemoglobin oxygen saturation in septic shock related to macrohemodynamic variables and outcome?. Crit. Care.

